# Functional Evaluation of Upper Urinary Tract with Diuretic Mercaptoacetyltriglycine Renal Scans in Patients with Benign Prostatic Obstruction before and after Surgical Intervention: A Pilot Study

**DOI:** 10.1155/2020/4605683

**Published:** 2020-08-10

**Authors:** Sung Yong Cho, Kyungtae Ko, Kyo Chul Koo, Hyung Joon Kim, Woo Jin Bang, Min Soo Choo, Sang Hyub Lee, Young Eun Yoon, Wonho Jung, Jae Young Choi, Dong Sup Lee, Young Endourological Study (YES) Group

**Affiliations:** ^1^Department of Urology, Seoul National University Hospital, Seoul, Republic of Korea; ^2^Department of Urology, Kangdong Sacred Heart Hospital, Hallym University, Seoul, Republic of Korea; ^3^Department of Urology, Gangnam Severance Hospital, Seoul, Republic of Korea; ^4^Department of Urology, Konyang University Hospital, Daejeon, Republic of Korea; ^5^Department of Urology, Hallym Sacred Heart Hospital, Hallym University, Anyang, Republic of Korea; ^6^Department of Urology, Dongtan Sacred Heart Hospital, Hallym University, Hwaseong, Republic of Korea; ^7^Department of Urology, KyungHee University Medical Center, Seoul, Republic of Korea; ^8^Department of Urology, Hanyang University Hospital, Seoul, Republic of Korea; ^9^Department of Urology, Dongsan Medical Center, Keimyung University School of Medicine, Daegu, Republic of Korea; ^10^Department of Urology, Yeungnam University Medical Center, College of Medicine, Yeungnam University, Daegu, Republic of Korea; ^11^Department of Urology, St. Vincent's Hospital, The Catholic University of Korea, Suwon, Republic of Korea; ^12^Endoluminal & Technology, Seoul, Republic of Korea

## Abstract

**Introduction:**

We investigated which benign prostatic hyperplasia-related lower urinary parameters are related to upper urinary tract obstruction and whether transurethral prostatectomy could improve upper urinary tract obstruction.

**Materials and Methods:**

Patients with prostate size over 30 g and urodynamically proven bladder outlet obstruction were enrolled in this prospective observational study. Bladder wall thickness and prostate size were measured by ultrasonography. A urodynamic study with laboratory tests including serum creatinine, prostate-specific antigen, and urinalysis was performed. Finally, a diuretic scintigraphy using mercaptoacetyltriglycine was performed. Tests except the urodynamic evaluation were repeated after transurethral prostatectomy.

**Results:**

In total, 24 patients were enrolled, and 19 patients completed the present study. The mean values of age (yrs), prostate size (mL), bladder thickness (mm), bladder compliance (*Δ*mL/*Δ*pr), and the bladder outlet obstruction index were 68.42 ± 8.25, 72.29 ± 32.78, 4.42 ± 1.14, 50.17 ± 32.15, and 82.11 ± 34.68, respectively. The mean T_1/2_ (min) was 17.51 ± 16.34 on the left side and 15.30 ± 11.96 on the right side. Statistical analysis showed that bladder compliance and bladder thickness were preoperatively related to upper urinary tract obstruction (*p* = 0.001 and *p* = 0.007, respectively). Diuretic mercaptoacetyltriglycine scan in 19 patients showed improvement 6 months after prostate surgery. Clinically significant proteinuria was associated with upper urinary tract obstruction, and proteinuria was also improved after prostate surgery.

**Conclusion:**

Storage-phase bladder dysfunction could be a reliable urodynamic factor for the indication of upper urinary tract obstruction in patients with benign prostatic hyperplasia, and upper urinary tract obstruction with subsequent kidney damage could be improved by surgical decompression of benign prostatic obstruction.

## 1. Introduction

Benign prostatic hyperplasia (BPH) is a prevalent disease in which approximately 50% of men aged 60 have lower urinary tract symptoms [1]. More importantly, BPH is a progressive disease [2] that may result in chronic renal insufficiency as one of the final stages of BPH progression [3]. Nevertheless, it is still unclear whether BPH is a risk factor for chronic kidney disease (CKD) regarding clinical evidence [4, 5]. If BPH causes chronic obstructive uropathy and eventually leads to renal insufficiency, there may be causative anatomical factors or urodynamic factors that could delay urinary drainage from the kidney to the urinary bladder.

With respect to the functional evaluation of upper urinary tract obstruction, the diuretic mercaptoacetyltriglycine (^99m^Tc-MAG3) renal scan (hereafter referred to as MAG3) is a feasible imaging tool to evaluate upper urinary tract obstruction [6]. To our knowledge, no clinical study has investigated the relationship of BPH-related lower urinary tract factors and upper urinary tract parameters with MAG3 in patients with BPH. Therefore, the authors conducted a pilot study to investigate whether benign prostatic obstruction (BPO)-related lower urinary tract parameters are related to upper urinary tract obstruction and whether the decompression of BPO could ameliorate upper urinary tract obstruction.

## 2. Materials and Methods

### 2.1. Study Design and the Enrollment of Patients

Patients who complained of lower urinary tract symptoms despite alpha blocker medication were prospectively enrolled from July 2016 to June 2019. Before enrollment, ultrasound (for kidney, bladder, and prostate; bladder thickness was measured at the bladder dome when patients felt the desire to voiding desire), a urodynamic study, and laboratory tests including PSA, serum creatinine with estimated glomerular filtration rate (eGFR) [7], and urinalysis were performed.

Patients with a PSA value of 4.0 (ng/mL) or higher underwent ultrasound-guided transrectal prostate biopsy to exclude prostate cancer. When eGFR was <60 ml/min/1.73 m^2^ or when clinically significant proteinuria was observed, the tests were repeated 3 months later to confirm CKD according to the universal protocol [8], and then, the better result was employed. Laboratory tests were repeated at 6 months after transurethral prostate surgery.

International Prostate Symptom Score (IPSS) questionnaire scores evaluated the following seven items were assessed: bladder emptying, frequency, intermittency, urgency, weak stream, straining, and nocturia [9]. The score from each item ranged from 0 to 5. The IPSS questionnaire was repeated 6 months after transurethral prostate surgery.

For noninvasive uroflowmetry, patients voided freely without catheterization. At that time, we measured and recorded the maximum flow rate (Qmax) and postvoid residual urine volume (PVR). The PVR was measured using a Biocon 500 ultrasound scanner (Medline Industries, Inc., Mundelein, Illinois, USA). Filling and voiding cystometry was performed, including parameters such as bladder compliance (*Δ*mL/*Δ*pr), the presence of involuntary detrusor contraction (IDC), the bladder outlet obstruction index (BOOI), Schäfer grades, and the bladder contractility index (BCI) [10]. A urodynamic study was performed only before surgery. Instead, patients were followed up with uroflowmetry 6 months postoperatively.

Patients who met all the following inclusion criteria (suggesting BPO) were enrolled in the present study: (1) those with evidence of BOO (BOOI ≥40 or Schäfer grade ≥2); and (2) those with a prostate total volume ≥30 mL in ultrasonography [11–13]. Patients who had any one of the following were excluded from the present study: (1) acute urinary retention during the screening period, (2) urinary tract infection during the screening period, (3) ongoing malignancy, (4) uncontrolled diabetes mellitus (HbA1c >8.0), (5) uncontrolled hypertension (systolic blood pressure >160 mmHg), (6) moderate or severe liver cirrhosis, (7) moderate or severe heart failure (NYHA class ≥ II), or (8) spinal cord disease such as myelopathy.

All patients enrolled in the study underwent diuretic MAG3. According to a previous investigation, 5 minutes was normal value of T_1/2_ in men aged 50~60 years [14]. The authors defined T_1/2_ = 10 minutes (time point when half of the ^99m^Tc substance is excreted) as the cut-off to distinguish whether the result of the renal scan was negative (<10 minutes) or positive (≥10 minutes), because (1) BPO patients with T_1/2_ >20 minutes would be very rare unless the obstruction was very severe and (2) 10 minutes is close to the median T_1/2_ value preoperatively. If the result was positive, the authors suggested it as “potential obstruction.” The MAG3 was postoperatively reevaluated at 6 months. We had also presented the perioperative change with the analysis of the continuous value of MAG3 to compensate the limitation of using the arbitrary cut-off level. Comparison between the mean preoperative MAG3 value and the mean postoperative MAG3 value and correlation between the change of MAG3 value and the other continuous values were performed.

To the best of our knowledge, the effect of transurethral prostate surgery for BPO on upper urinary tract drainage has never been reported. The authors analyzed the effect of the transurethral prostate surgery for BPO on the drainage condition as follows. If a 25% or more decrease (at least 1 kidney) was observed in the T_1/2_ of the postoperative result compared to the T_1/2_ of the preoperative result, the authors assumed that the upper urinary tract drainage was “relatively” enhanced; furthermore, if a 50% or more decrease (at least 1 kidney) was identified in the T_1/2_ of MAG3, the authors assumed the upper urinary tract drainage was “definitely” enhanced.

### 2.2. Transurethral Prostate Surgery

Transurethral prostate surgery was performed by surgeons with experience in over 50 cases of holmium laser enucleation of the prostate. Laser energy for enucleation of an adenoma was generally 2.0 J with 20 Hz. The other techniques were similar to those in previously reported methods [15].

### 2.3. Statistics

The authors hypothesized that the upper urinary tract drainage measured by MAG3 could be enhanced 6 months after transurethral surgery in BPO. As a pilot study, two-tailed sign test (binominal test) was used with a beta error of 0.2, an alpha error of 0.05, and an effect size as 0.3 to determine that the target sample size was 20 patients. Assuming a drop-out rate of approximately 20%, we enrolled 24 patients in the present study.

The Mann–Whitney *U* test and Wilcoxon test were used to check the difference before and 6 months after operation. The chi-squared test or Fisher's exact test was used to analyze binominal relationships between the groups. Spearman's test was used to investigate the association among the parameters. All statistical analyses were performed with the statistical package IBM-SPSS for Windows (Version 23.0; IBM Crop, Armonk, NY, USA). *p* < 0.05 was regarded as statistically significant.

## 3. Results

In total, 24 patients were enrolled in the present study. Five patients were excluded due to the postoperative confirmation of pathologically-proven prostate cancer in 3 patients, loss to follow-up in one patient, and cervical myelopathy discovered during follow-up in 1 patient. In total, 19 patients completed the study.

The mean age was 68.42 ± 8.25 years, and the mean BMI was 23.83 ± 1.66 kg/m^2^. The baseline patient characteristics are shown in Table 1.

Among 19 patients with BPO, a preoperative T_1/2_ of MAG3 over 10 minutes (potential obstruction) was observed in 13 patients (21 kidneys). Preoperative potential obstruction in at least 1 kidney was associated with clinically significant proteinuria (Spearman′s rho = 0.482, *p* = 0.036). The associations between preoperative potential obstruction in at least 1 kidney and other factors are shown in Table 2.

At 6 months after transurethral prostate surgery, potential obstruction remained in 6 patients (9 kidneys). Based on the aforementioned criteria, “relative” improvement of upper urinary tract drainage was observed in 16 patients (26 kidneys), and “definite” improvement was seen in 4 patients (6 kidneys).

**Figure 1:**
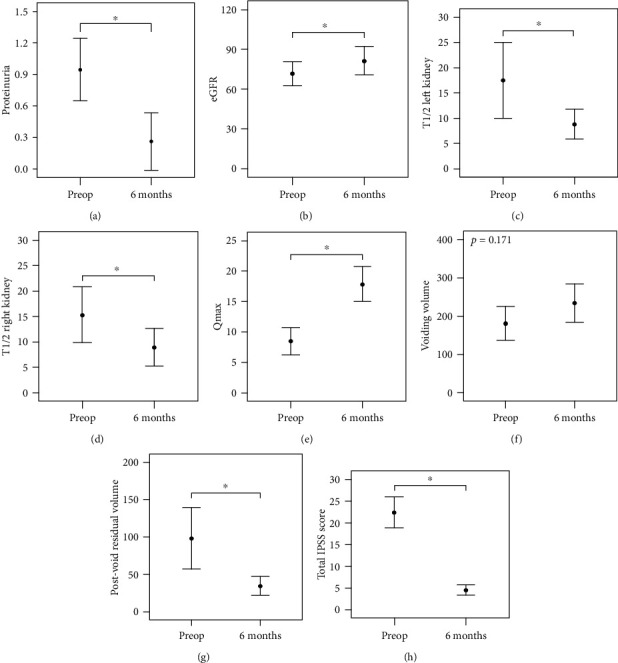
Changes in parameters before and at 6 months after the decompression of BPO. (a, b) Renal function was improved. (c, d) The T_1/2_ of diuretic ^99m^Tc-MAG3 was improved. (e–g) The mean values of Qmax, voiding volume, and PVR seemed to be enhanced, but voiding volume did not significantly increase. (h) The IPSS score was markedly improved. BPO: benign prostatic obstruction; PVR: postvoid residual urine volume; IPSS: international prostate symptom score. Asterisk (^∗^) means *p* < 0.05.

All laboratory tests, including prostate-specific antigen, eGFR, and urinary proteinuria, were improved after transurethral prostate surgery. The mean prostate-specific antigen level decreased from 6.67 ± 4.52 to 1.55 ± 1.44. The mean QoL scores in the IPSS questionnaire improved from 4.78 ± 0.73 to 1.11 ± 0.76. The other results from the comparison between preoperative values and postoperative values are shown in Figure 1. Although voiding volume did not increase significantly, Qmax was remarkably improved, and postvoid residual volume decreased obviously.

**Figure 2:**
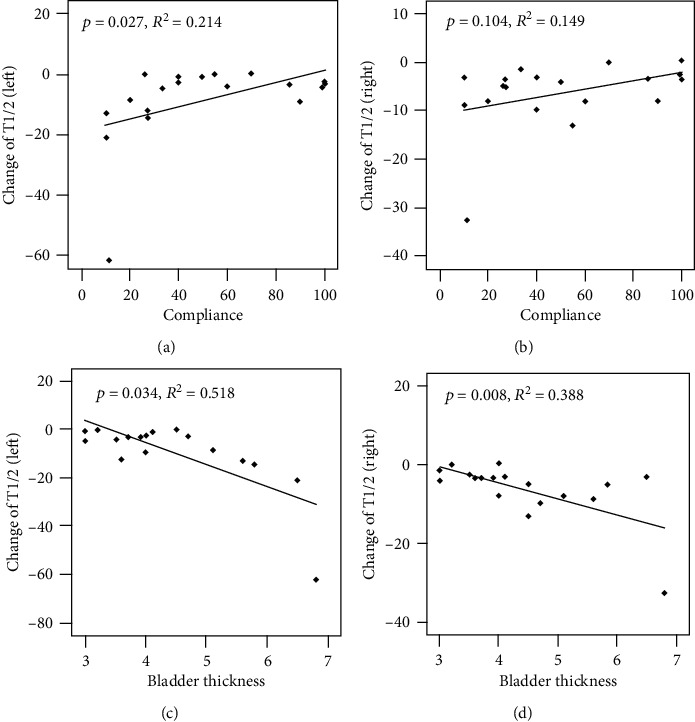
Changes in the T_1/2_ of the diuretic ^99m^Tc-MAG3 scan according to preoperative bladder compliance and bladder thickness. (a, b) The preoperative bladder compliance was lower, and the change in the T_1/2_ of the diuretic ^99m^Tc-MAG3 scan was greater. (c, d) The preoperative bladder was thicker, and the change in the diuretic ^99m^Tc-MAG3 scan was greater.

**Figure 3:**
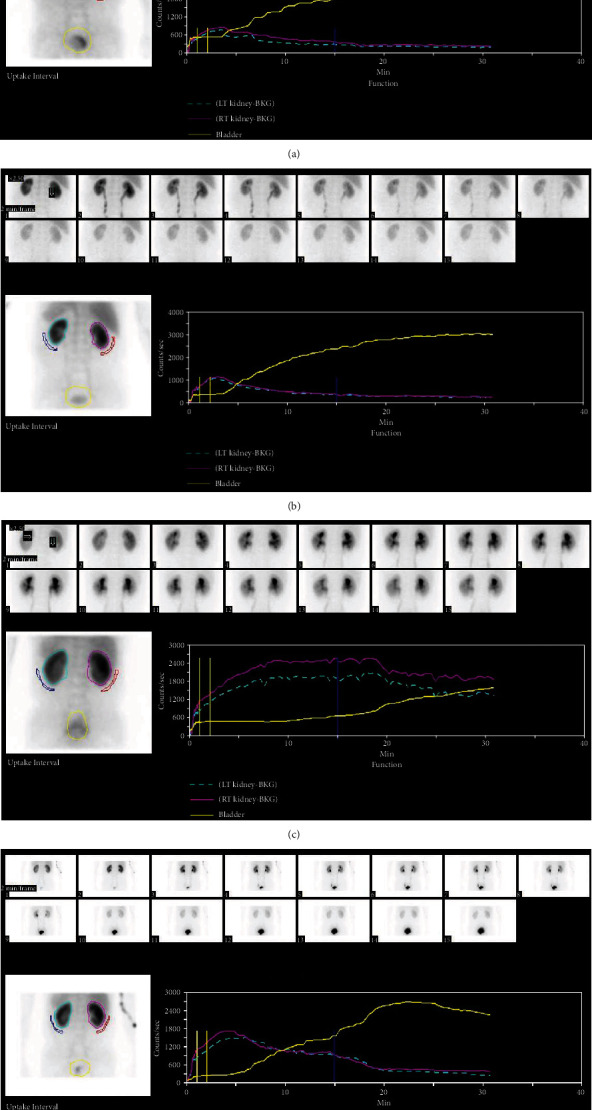
Changes in the T_1/2_ of the diuretic ^99m^Tc-MAG3 scan according to the preoperative T_1/2_ of the diuretic ^99m^Tc-MAG3 scan results. (a, b) Preoperative values of the T_1/2_ of the diuretic ^99m^Tc-MAG3 scan were 7.0 in the left kidney and 9.3 in the right kidney, which means no delay in the T_1/2_ of the upper urinary tract (a). The values were improved to 4.7 in the left kidney and 5.3 in the right kidney postoperatively (b). (c, d) Preoperative values of the T_1/2_ of the diuretic ^99m^Tc-MAG3 scan were 73.3 in the left kidney and 44.0 in the right kidney, which means obvious upper urinary tract obstruction (c). The values were markedly improved to 11.6 in the left kidney and 11.6 in the right kidney postoperatively (d).

As lower urinary factors such as bladder compliance and bladder thickness were related to the T_1/2_ of MAG3, the authors investigated the likelihood of improvement in the T_1/2_ of MAG3 based on bladder compliance and bladder thickness. Because most patients with preoperatively good compliance (less thickened bladder) had a T_1/2_ of MAG3 under 10 minutes, the value did not change very much at 6 months after operation, whereas BPO patients who had preoperatively low compliance with thickened bladder and a tendency for delay in T_1/2_ had much room for improvement in the T_1/2_ of MAG3 (Figures 2 and 3). This observation means that transurethral prostate surgery in BPO patients with low compliance and/or thickened bladder may be beneficial to improve upper urinary tract drainage.

Clinically, significant proteinuria remained in 4 patients (preoperative proteinuria was found in 15 patients), and potential obstruction was still observed in 6 patients (preoperative potential obstruction was observed in 13 patients) at 6 months after the operation. In the correlation test, postoperative proteinuria was related not to upper urinary tract obstruction but to underlying diabetes mellitus (Table 3).

## 4. Discussion

The interesting finding of this study is that filling-phase bladder dysfunction, such as low compliance, could explain the occurrence of obstruction in the MAG3 in the present cohort. A similar study was conducted with pediatric neurogenic bladders, where the authors emphasized low bladder compliance and bladder wall thickness as a predictor of renal damage [16]. Many clinical studies support the likelihood of an association between bladder wall thickness and BPO [17], describing that the measurement of bladder wall thickness has a median sensitivity of 82% and a specificity of 92% for the prediction of BOO. De Nunzio et al. found that detrusor overactivity was significantly associated with bladder wall thickness even in non-BPO patients [18]. Therefore, the thickened bladder is not an exclusive property of BOO (or BPO); rather, it may be more related to detrusor condition regardless of BPO. Consequently, chronic BOO secondary to BPH can be a nonneurogenic factor among various lower urinary tract conditions that thicken the urinary bladder wall [19].

The BOOI was not significantly associated with the status of the upper urinary tract drainage measured by the T_1/2_ of MAG3. Instead, only bladder compliance was responsible for upper urinary tract drainage in the present study. Two cases of IDC also showed a delay in the T_1/2_ of MAG3. These findings indicated that BOO itself would not induce obstruction in the upper urinary tract, but the long-standing high intravesical pressure induced by storage-phase bladder dysfunction might be the reason for upper urinary tract obstruction. Although the number of patients included is small, the authors suggest that BPH might not be the direct cause of renal insufficiency and BPH may be one of the factors that may induce bladder dysfunction, such as low compliance, which can affect urine drainage. This may also mean that the transient elevation of intravesical pressure during voiding in patients with BPH or a high BOOI without abnormalities in the filling phase would not induce renal insufficiency. This finding is consistent with previous investigations with neurogenic bladder [20, 21].

Considering that PdetQmax is incorporated in the BCI and the BOOI, it is possible that detrusor contraction increased to overcome the large size of the prostate. Nevertheless, the BCI was also not related to the T_1/2_ of MAG3. Because evidence of BOO (with a BOOI of 40 or more) was a criterion for inclusion in the present study, the bladder contractility in the majority of patients was intact or relatively preserved. According to several suggested criteria for detrusor underactivity (DU) [22, 23], PdetQmax should be under 30 cmH_2_O. Therefore, no one could have had DU in this cohort. However, researchers should be aware that either BPO without DU or impaired detrusor activity can cause chronic urinary retention (CUR) [24], and CUR can be associated with hydronephrosis [25]. CUR is a clinical definition (not by urodynamic study), and most of the literature indicates CUR as PVR over 300 mL [25]. In the present study, only 1 case of CUR was shown in a patient with a prostate size of 58.5 mL, BOOI of 48, BCI of 55, bladder compliance of 33, and T_1/2_ of MAG3 over 10 minutes in the left kidney. It indicates that the patient had high-pressure CUR (BPO without DU) [26]. Therefore, one of the limitations of the present study is that the authors cannot determine whether DU in BPH patients (BPH with low-pressure CUR) could also indicate a connection between bladder compliance and a delay in the T_1/2_ of MAG3 in terms of CUR.

Prostate size was not associated with the T_1/2_ of MAG3, as the BOOI and BCI were not related to the T_1/2_ of MAG3. Previous studies have already suggested that prostate size might not be related to the decrease in renal function [5, 27]. Consequently, the authors believe that prostate size and relevant functional parameters, such as the BOOI and/or BCI, could not directly influence the delay in urinary drainage from the upper urinary tract because the urinary bladder acts as a “buffer.” In the case of a loss of flexibility of the buffer (low compliance) or of a fullness of the buffer (CUR), the end-organ (BPO) effect could develop. Therefore, preoperative urodynamic evaluation in BPH is very important.

It was very interesting that preoperative proteinuria was associated with a delay in the T_1/2_ of MAG3. A recent study showed that clinically significant proteinuria was more frequently identified in BPO patients with low bladder compliance [28], and the authors found that transurethral prostate surgery for those patients mitigated proteinuria and that the phenomenon was obvious in patients without underlying medical disease. Considering that proteinuria is a marker of ongoing renal damage [29], the present study revealed the probability that BPO with functionally low bladder compliance and an anatomically thickened bladder wall may delay the T_1/2_ of MAG3, resulting in renal damage (proteinuria) even in patients without CUR.

The postoperative T_1/2_ of MAG3 was not related to the remaining proteinuria at 6 months postoperatively, which indicated that underlying hypertension and/or DM might be more responsible for the remaining proteinuria (Table 3). Conversely, BPO could be involved in the pathophysiology of developing CKD. Considering CKD can also be multifactorial, we could not definitely rule out intrinsic renal causes presenting proteinuria because we did not perform or recommend renal biopsy in the present cohort, which could be a limitation of the study. Nevertheless, in the present study, proteinuria, eGFR, and the T_1/2_ of MAG3 were significantly improved after surgery. The authors think that the preoperative discordance between eGFR and T_1/2_ of MAG3 might be due to the following reasons: (1) the baseline eGFRs were close-set one another; (2) individual baseline eGFR could be determined not only by the urinary drainage condition but also by various factors such as age and underlying diseases including hypertension and DM; and (3) the decrease in eGFR is a quite late response compared to the development of significant proteinuria [30].

Nomura et al. showed urodynamic results in patients who underwent transurethral prostate surgery. Half of those patients with detrusor overactivity improved 6 months after operation [31]. Furthermore, an interesting result was reported by Tkocz et al. that transurethral resection or incision of the prostate could sufficiently improve bladder compliance as well as remarkably decrease the incidence of detrusor overactivity unless the detrusor structure had irreversibly changed [32]. The absence of postoperative urodynamic analysis was a major limitation of the present study, but the preoperative relationship between the T_1/2_ of MAG3 and bladder compliance and the improvement in the T_1/2_ of MAG3 could support our assumption for the improvement in postoperative bladder compliance.

Meanwhile, 5 alpha reductase inhibitors (5ARIs) can be used for pharmacological deobstruction for BPO. According to the early research conducted by Stoner who used finasteride for BPH treatment for 36 months, he found that the prostate volume was reduced by 27% compared to baseline (over 45 mL), and Qmax was improved by 2.3 mL/s [33]. Similarly, in CombaT study, even with 24 months of combination therapy (dutasteride plus tamsulosin), Qmax improved by 2.2~3.0 mL/s [34]. Although pharmacological deobstruction using 5ARIs (with or without alpha 1 selective adrenoreceptor antagonist) could be beneficial to relieve the BPO, (1) it takes too much time to observe the results, and (2) the change of parameters such as increase in Qmax and decrease in prostate volume may be tiny, compared to surgical decompression. Therefore, the pharmacological deobstruction seems to be inappropriate as a control group of a study evaluating the MAG3 parameter.

Finally, the present study contains severe forms of BPH, with a mean bladder compliance, BOOI, prostate size, and IPSS score of 50.17, 82.11, 72.29, and 22.44, respectively, which cautions that the data of this cohort cannot be generalized for all BPH patients. However, the present study showed the importance of bladder function, which was central to the explanation of the probability of CKD progression in BPO patients, and provided a message of the importance of decompression of BPO in the prevention of kidney damage due to the delay in urinary drainage.

## 5. Conclusions

In conclusion, storage-phase bladder dysfunction (low compliance and/or detrusor overactivity) may be a key factor for kidney damage resulting from delayed urinary drainage in patients with BPO. Such a delay could be improved by the decompression of BPO. Based on the results of the present study with supporting literature, the authors suggest that the role of BPH progression in the development of CKD is as follows: “BPH ➔ BOO ➔ storage-phase bladder dysfunction with bladder wall thickness ➔ delay in upper urinary tract drainage ➔ kidney damage (proteinuria) ➔ CKD progression”. Therefore, when surgeons encounter patients with severe BPH, they should seriously consider preoperative urodynamic studies to evaluate storage-phase bladder function. Given the cost of diuretic renal scans, surgeons should also perform urinalysis in those patients to detect proteinuria before and after surgery.

## Figures and Tables

**Table 1:** Patients' baseline characteristics (*n* = 19).

Age (years)	68.42 ± 8.25
Body mass index (kg/m^2^)	23.83 ± 1.66
Prostate-specific antigen (ng/mL)	6.67 ± 4.52
Serum creatinine (mg/dL)	1.25 ± 0.90
eGFR (mL/min/1.73m^2^)	71.68 ± 19.43
Proteinuria	
Trace or more	15/19 (78.9%)
+1 or more	3/19 (15.8%)
Uroflowmetry parameters	
Qmax (mL/sec)	8.51 ± 4.91
Voiding volume (mL)	181.11 ± 96.74
Postvoid residual volume (mL)	98.53 ± 89.92
Invasive urodynamic parameters	
Bladder compliance (*Δ*mL/*Δ*pr)	50.17 ± 32.15
Involuntary detrusor contraction	2/19 (10.5%)
Bladder outlet obstruction index	82.11 ± 34.68
Schäfer grades	3.95 ± 1.47
Bladder contractility index	114.45 ± 40.24
Anatomical factors	
Prostate size (mL)	72.29 ± 32.78
Bladder thickness (mm)	4.42 ± 1.14
IPSS total score	22.44 ± 7.51
Storage symptom score	8.72 ± 3.88
Obstructive symptom score	13.72 ± 4.03
Quality of life score	4.78 ± 0.73
T_1/2_ of ^99m^Tc-MAG3	
Left side	17.51 ± 16.34
Right side	15.30 ± 11.96
T_1/2_ >10 min at least one kidney	13/19 (68.4%)
Underlying conditions	
Diabetes mellitus	4/19 (21.1%)
Hypertension	7/19 (36.8%)
Smoking	5/19 (26.3%)
Under 20 p^∗^yrs	2/19 (10.5%)
Over 20 p^∗^yrs	3/19 (15.8%)
Education (high school or more)	13/19 (68.4%)
Recent symptom aggravation (year)	1.33 ± 1.09
Previous history of acute urinary retention	3/19 (15.8%)

Parameters were expressed by the mean ± standard deviation or frequency (%). eGFR: estimated glomerular filtration rate; Qmax: maximal flow rate; IPSS: international prostate symptom score; MAG-3: diuretic mercaptoacetyltriglycine.

**Table 2:** Association between potential obstruction^1^ and other factors in preoperative data.

Upper tract factor	
Clinically significant proteinuria	0.482, *p* = 0.036^∗^
eGFR	0.083, *p* = 0.736
Lower tract factor	
Bladder thickness	0.614, *p* = 0.007^∗^
Prostate size	-0.269, *p* = 0.266
Urodynamic factor	
Storage factors	
Voiding volume	-0.310, *p* = 0.196
Bladder compliance	-0.704, *p* = 0.001^∗^
Obstructive factors	
Qmax	-0.021, *p* = 0.933
Postvoid residual volume	-0.145, *p* = 0.554
Bladder outlet obstruction index	0.041, *p* = 0.866
Other factor	
Bladder contractility index	-0.031, *p* = 0.583
General factors	
Age	-0.031, *p* = 0.899
Body mass index	-0.165, *p* = 0.499
Diabetes mellitus	-0.205, *p* = 0.401
Hypertension	-0.049, *p* = 0.841

Data were expressed by Spearman's rho with *p* value. ^∗^*p* < 0.05. ^1^: potential obstruction means preoperative T_1/2_ of ^99m^Tc-MAG3 >10 minutes at least 1 kidney.

**Table 3:** Association between postoperative significant proteinuria and other factors in postoperative data.

Upper tract factor	
Potential obstruction^1^	0.233, *p* = 0.338
eGFR	-0.313, *p* = 0.191
Lower tract factor	
Uroflowmetry	
Qmax	-0.093, *p* = 0.706
Voiding volume	-0.054, *p* = 0.825
Postvoid residual volume	-0.440, *p* = 0.059
General factors	
Age	0.118, *p* = 0.630
Body mass index	-0.163, *p* = 0.505
Diabetes mellitus	0.696, *p* = 0.001^∗^
Hypertension	0.420, *p* = 0.073

Data were expressed by Spearman's rho with *p* value. ^∗^*p* < 0.05. ^1^: potential obstruction means postoperative T_1/2_ of ^99m^Tc-MAG3 >10 minutes at least 1 kidney.

## Data Availability

The data used to support the findings of this study are available from the corresponding author upon request.

## Supplementary Materials

Supplementary Materials . The Exel data used to support the findings of this study have been deposited in the Supplementary file.
